# Reply to Skokou, M. Comment on “Keeler et al. Ketamine as a Treatment for Anorexia Nervosa: A Narrative Review. *Nutrients* 2021, *13*, 4158”

**DOI:** 10.3390/nu14102119

**Published:** 2022-05-19

**Authors:** Johanna Louise Keeler, Janet Treasure, Mario F. Juruena, Carol Kan, Hubertus Himmerich

**Affiliations:** 1 Section of Eating Disorders, Department of Psychological Medicine, Institute of Psychiatry, Psychology & Neuroscience, King’s College London, London SE5 8AF, UK; janet.treasure@kcl.ac.uk (J.T.); hubertus.himmerich@kcl.ac.uk (H.H.); 2 South London and Maudsley NHS Foundation Trust, Bethlem Royal Hospital, Monks Orchard Road, Beckenham BR3 3BX, UK; mario.juruena@kcl.ac.uk; 3 Centre for Affective Disorders, Department of Psychological Medicine, Institute of Psychiatry, Psychology & Neuroscience, King’s College London, London SE5 8AF, UK; 4 Eating Disorder Service, Central and North West London NHS Foundation Trust, 1 Nightingale Place, Kensington & Chelsea, London SW10 9NG, UK; carol.kan@nhs.net

In response to our narrative review, which suggested the use of the glutamatergic n-methyl-D-aspartate (NMDA) receptor antagonist ketamine as a potential treatment for anorexia nervosa (AN) [1], Maria Skokou posed the question whether other glutamatergic medications (e.g., lamotrigine) might be effective in the treatment of eating disorders in general [2]. Therefore, we would like to develop the idea further that the glutamate system might be of relevance for the pathophysiology and the treatment of eating disorders.

Glutamate is the most common neurotransmitter in the central nervous system, as it is present in more than 80% of synapses in the brain. Glutamate unfolds its excitatory effects at synaptic and non-synaptic receptors on the membranes of neuronal and glial cells. Its receptors are important for synaptic transmission, plasticity, and development, learning and memory [3]. They can be categorised into voltage-sensitive ionotropic receptors such as NMDA, kainate, and alpha-amino-3-hydroxyl-5-methyl-4-isoxazole-propionate (AMPA) receptors and ligand-sensitive metabotropic glutamate receptors (mGluRs) [4]. Changes in glutamatergic signalling have been implicated in the pathophysiology of major psychiatric disorders such as depression, schizophrenia and autism spectrum disorder [5]. Glutamate receptors have specifically been suggested to be involved in the development of eating disorder symptoms. NMDA receptors have been implicated in the control of appetite and food preference [6]; and alterations in mGluRs, NMDA and AMPA receptors have been related to addictive behaviours towards food [7,8].

Various drugs and medications influence glutamate signalling including the following: the stimulant modafinil; the anaesthetic ketamine; the anxiolytic and antibiotic drug d-cycloserine; the novel antipsychotic lumateperone, antiepileptic drugs such as lamotrigine and topiramate; the antidementive drug memantine; the anti-craving medication acamprosate (e.g., [9]).

Some of these drugs have been tested in people with eating disorders, e.g., d-cycloserine, which is a partial agonist at the glycine recognition site of the glutamatergic NMDA receptor, as well as topiramate and lamotrigine, which both decrease glutamate release and signalling [10].

For d-cycloserine, we have conflicting results from small studies. Steinglass and colleagues [11] tested the administration of d-cycloserine before meal-exposure in AN. However, caloric intake did not increase. A similar trial by Levinson and colleagues [12] with a one-month follow-up resulted in an increase in body mass index under d-cycloserine compared to the placebo group.

Skokou [2] already mentioned the small case series which tested lamotrigine in people with bulimia nervosa (BN) [13,14], where lamotrigine treatment was associated with reductions in eating disorder symptoms. People with BN often experience problems with their mood, affect and impulsiveness. A recent study showed that the combination of lamotrigine and dialectical behaviour therapy (DBT) helps patients with bulimic-spectrum eating disorders regarding affective lability and impulsive behaviour [15]; in addition, it has been suggested that lamotrigine is helpful in co-morbid bipolar depression in people with BN and binge eating disorder (BED) [16].

However, the currently most promising glutamatergic agent in eating disorders seems to be topiramate. Two independent RCTs [17,18,19] showed superiority of topiramate to placebo in reducing the frequency of binge eating episodes and compensatory measures (e.g., vomiting and use of laxatives) in patients with BN. In BED, a large multi-centre, RCT with 407 patients [20] showed that topiramate significantly reduces binge eating frequency, leads to weight loss and improves BED symptoms compared to placebo. Two smaller RCTs [21,22] yielded similar findings. AN is a contraindication for topiramate though, because it leads to weight loss.

Taken together, we agree with Skokou [2] that the glutamate system might be highly relevant for the treatment of eating disorders. As explained in our previous paper [1], we particularly believe that ketamine might lead to a breakthrough in the pharmacological treatment of AN. Additionally, glutamatergic agents may help with psychiatric co-morbidities of people with eating disorders.

## References

[1] Keeler J.L., Treasure J., Juruena M.F., Kan C., Himmerich H. (2021). Ketamine as a Treatment for Anorexia Nervosa: A Narrative Review. Nutrients.

[2] Skokou M. (2022). Comment on Keeler et al. Ketamine as a Treatment for Anorexia Nervosa: A Narrative Review. *Nutrients* 2021, *13*, 4158. Nutrients.

[3] Riedel G., Platt B., Micheau J. (2003). Glutamate receptor function in learning and memory. Behav. Brain Res..

[4] Stevens L., Rodin I., Stevens L., Rodin I. (2011). Introduction to Drug Treatments. Psychiatry.

[5] Olloquequi J., Cornejo-Córdova E., Verdaguer E., Soriano F.X., Binvignat O., Auladell C., Camins A. (2018). Excitotoxicity in the pathogenesis of neurological and psychiatric disorders: Therapeutic implications. J. Psychopharmacol..

[6] Sasaki T., Matsui S., Kitamura T. (2016). Control of appetite and food preference by NMDA receptor and its co-agonist d-serine. Int. J. Mol. Sci..

[7] Hadad N.A., Knackstedt L.A. (2014). Addicted to palatable foods: Comparing the neurobiology of Bulimia Nervosa to that of drug addiction. Psychopharmacology.

[8] Yohn S.E., Galbraith J., Calipari E.S., Conn P.J. (2019). Shared Behavioral and Neurocircuitry Disruptions in Drug Addiction, Obesity, and Binge Eating Disorder: Focus on Group I mGluRs in the Mesolimbic Dopamine Pathway. ACS Chem. Neurosci..

[9] Olive M.F., Cleva R.M., Kalivas P.W., Malcolm R.J. (2012). Glutamatergic medications for the treatment of drug and behavioral addictions. Pharmacol. Biochem. Behav..

[10] Czapinski P., Blaszczyk B., Czuczwar S.J. (2005). Mechanisms of action of antiepileptic drugs. Curr. Top. Med. Chem..

[11] Steinglass J., Sysko R., Schebendach J., Broft A., Strober M., Walsh B.T. (2007). The application of exposure therapy and D-cycloserine to the treatment of anorexia nervosa: A preliminary trial. J. Psychiatr. Pract..

[12] Levinson C.A., Rodebaugh T.L., Fewell L., Kass A.E., Riley E.N., Stark L., McCallum K., Lenze E.J. (2015). D-Cycloserine facilitation of exposure therapy improves weight regain in patients with anorexia nervosa: A pilot randomized controlled trial. J. Clin. Psychiatry.

[13] Trunko M.E., Schwartz T.A., Berner L.A., Cusack A., Nakamura T., Bailer U.F., Chen J.Y., Kaye W.H. (2017). A pilot open series of lamotrigine in DBT-treated eating disorders characterized by significant affective dysregulation and poor impulse control. Bord. Personal. Disord. Emot. Dysregulation.

[14] Trunko M.E., Schwartz T.A., Marzola E., Klein A.S., Kaye W.H. (2014). Lamotrigine use in patients with binge eating and purging, significant affect dysregulation, and poor impulse control. Int. J. Eat. Disord..

[15] Reilly E.E., Berner L.A., Trunko M.E., Schwartz T., Anderson L.K., Krueger A., Yu X., Chen J.Y., Cusack A., Nakamura T. (2022). Evaluating the use of lamotrigine to reduce mood lability and impulsive behaviors in adults with chronic and severe eating disorders. Eat. Weight. Disord. Stud. Anorex. Bulim. Obes..

[16] Himmerich H., Kan C., Au K., Treasure J. (2021). Pharmacological treatment of eating disorders, comorbid mental health problems, malnutrition and physical health consequences. Pharmacol. Ther..

[17] Hedges D.W., Reimherr F.W., Hoopes S.P., Rosenthal N.R., Kamin M., Capece J.A. (2003). Treatment of bulimia nervosa with topiramate in a randomized, double-blind, placebo-controlled trial, part 2: Improvement in psychiatric measures. J. Clin. Psychiatry.

[18] Hoopes S.P., Reimherr F.W., Hedges D.W., Rosenthal N.R., Kamin M., Karim R., Capece J.A., Karvois D. (2003). Treatment of bulimia nervosa with topiramate in a randomized, double-blind, placebo-controlled trial, part 1: Improvement in binge and purge measures. J. Clin. Psychiatry.

[19] Nickel C., Tritt K., Muehlbacher M., Pedrosa Gil F., Mitterlehner F.O., Kaplan P., Lahmann C., Leiberich P.K., Krawczyk J., Kettler C. (2005). Topiramate treatment in bulimia nervosa patients: A randomized, double-blind, placebo-controlled trial. Int. J. Eat. Disord..

[20] McElroy S.L., Hudson J.I., Capece J.A., Beyers K., Fisher A.C., Rosenthal N.R., Group T.B.E.D.R. (2007). Topiramate for the treatment of binge eating disorder associated with obesity: A placebo-controlled study. Biol. Psychiatry.

[21] Claudino A.M., de Oliveira I.R., Appolinario J.C., Cordás T.A., Duchesne M., Sichieri R., Bacaltchuk J. (2007). Double-blind, randomized, placebo-controlled trial of topiramate plus cognitive-behavior therapy in binge-eating disorder. J. Clin. Psychiatry.

[22] McElroy S.L., Arnold L.M., Shapira N.A., Keck P.E., Rosenthal N.R., Karim M.R., Kamin M., Hudson J.I. (2003). Topiramate in the treatment of binge eating disorder associated with obesity: A randomized, placebo-controlled trial. Am. J. Psychiatry.

